# circRNA-002178 act as a ceRNA to promote PDL1/PD1 expression in lung adenocarcinoma

**DOI:** 10.1038/s41419-020-2230-9

**Published:** 2020-01-16

**Authors:** JunFeng Wang, XuHai Zhao, YanBo Wang, FengHai Ren, DaWei Sun, YuBo Yan, XiangLong Kong, JianLong Bu, MengFeng Liu, ShiDong Xu

**Affiliations:** Harbin Medical University Cancer Hospital, Harbin, Heilongjiang China

**Keywords:** Non-small-cell lung cancer, Oncogenes

## Abstract

Circular RNAs (circRNAs) have been identified play a vital role in various different types of cancer via sponging miRNAs (microRNAs). However, their role in lung adenocarcinoma (LUAD) remains largely unclear. In this study, we systematically characterized the circRNA expression profiles in the LUAD cancer tissues and paired adjacent non-cancerous tissues. Three circRNAs were found to be significantly upregulated. Among them, has-circRNA-002178 was further confirmed to be upregulated in the LUAD tissues, and LUAD cancer cells. Subsequently, we also found has-circRNA-002178 could enhance PDL1 expression via sponging miR-34 in cancer cells to induce T-cell exhaustion. More importantly, circRNA-002178 could be detected in exosomes of plasma from LUAD patients and could serve as biomarkers for LUAD early diagnosis. Finally, we found circRNA-002178 could be delivered into CD8^+^ T cells to induce PD1 expression via exosomes. Taken together, our study revealed that circRNA-002178 could act as a ceRNA to promote PDL1/PD1 expression in lung adenocarcinoma.

## Introduction

Lung cancer is the leading cause of cancer-related death worldwide^1^. According to histologic types and prognosis, lung adenocarcinoma (LUAD) accounts for ~50% of all types of lung cancer and is increasing year by year, especially in women and young adults^1^. Despite the improvements of diagnosis and treatment, the 5-year survival rate for LUAD is <20%^1^. Therefore, it is urgently needed to find novel early diagnosis biomarkers and treatment targets for LUAD.

More than 90% human transcripts are found limited protein-coding capacity, but encoded non-coding RNA, including microRNA (miRNA), long non-coding RNA (lncRNA), and circular RNA (circRNA)^2^. CircRNAs are derived from precursor mRNA back-splicing with a circular covalently closed structure and higher tolerance to exonuclease digestion^3^. During the past few years, thousands of circRNAs have been identified in various organisms and found play a key role in many diseases, especially cancer^3^. Accumulating evidence is now unveiling that circRNAs could act as ceRNA by sponging miRNA to relieve the repression of miRNAs for their targets. Exosomes are cell-derived vesicles with diameters of 30–100 nm. Studies have confirmed exosomes are present in many body fluids, including blood, urine, and saliva^4^. CircRNA have been found to be stably present in exosomes, and could serve as novel non-invasive biomarkers^5^. More interestingly, these circRNAs present in the exosomes could act as messengers in cell-to-cell communications to regulate the function of target cells^6^.

Tumors cells could grow and metastasize by avoiding recognition and attack by the immune system (which is named tumor immune escape) through various mechanisms, such as the expression of Programmed death-ligand 1 (PDL1, also called CD274, B7-H1)^7^. PDL1, 40-kDa transmembrane protein, has been found to be abundantly expressed in almost all kinds of cancer cells and involved in tumor immune escape through the interaction with programmed cell death protein 1 (PD1)^8^. PD1, encoded by the PDCD1 gene, is a type I transmembrane protein composed of 288 amino acid residues, belonging to the immunoglobulin CD28 family. Studies have confirmed PD1 can activate intracellular signaling pathways to inhibit the activation of immune cells or exhaust the immune cell, especially activated T cells in tumor condition to help cancer cells escape T-cell-mediated death and resist anti-tumor immune responses^8^. Blocking the PDL1/PD1 pathway is recognized as a very promising approach for tumor treatment of tumor^8^. However, the upstream regulatory mechanism of PDL1 and PD1 still remains largely unknown.

In this study, the circRNAs of LUAD cancer tissues and paired adjacent non-cancerous tissues was characterized by circRNA microarray profile and quantitative reverse transcription PCR (qRT-PCR). CircRNA-002178 was found to be upregulated in LUAD tissues. Subsequently, we found has-circRNA-002178 could enhance PDL1 expression via sponging miR-34 in cancer cells by function assay. Moreover, we also found CircRNA-002178 exist in exosomes of plasma from LUAD patients and could serve as biomarkers for LUAD early diagnosis. Additionally, CircRNA-002178 in the exosomes could be delivered into T cells to promote PD1 expression via sequestering miR-28-5p. In conclusion, our results suggested that has-circRNA-002178 could be used as a potential non-invasive biomarker for the early detection of LUAD, and function as a ceRNA to enhance PDL1 and PD1 expression in cancer cells and T cells, respectively.

## Methods

### Patients’ characteristics

The expression profile of circRNAs in LUAD was downloaded from the GEO database (GSE101684 and GSE101586). The 105 pairs of LUAD tissues and adjacent non-cancerous tissues were collected from patients who were diagnosed with LUAD at the Harbin Medical University Cancer Hospital (Harbin, China). The serum samples from 30 healthy volunteers and 120 LUAD patients without any treatment were collected at the Harbin Medical University Cancer Hospital (Harbin, China). The detailed clinicopathological features are described in Table 1. The written informed consent was obtained from all patients. The study was authorized by the Ethics Committee of the Harbin Medical University Cancer Hospital and conducted in conformity to the Declaration of Helsinki.

**Table 1:** Patient characteristics and clinical features of LUAD tissues.

Variable	Plasma			Tissue
		LUAD	Normal	LUAD
		(*n* = 120)	(*n* = 30)	(*n* = 105)
Average age (years)		61.3 ± 7.9	62.7 ± 4.6	61.2 ± 8.5
Gender	Female	64	15	63
	Male	56	15	42
Stage	I	60		27
	II	30		41
	III	25		37
	IV	5		0

### Cell culture

Human LUAD cell lines A549, PC9, and 95D, and normal human bronchial epithelial cell line BEAS-2B were purchased from American Type Culture Collection (ATCC) (Manassas, VA, USA). BEAS-2B was cultured in RPMI 1640 medium supplemented with 10% fetal bovine serum (FBS) (HyClone, Logan, UT, USA), penicillin (10,000 units/ml), and streptomycin (10,000 μg/ml) (Invitrogen). A549, PC9, and 95D were cultured in Dulbecco’s Modified Eagle’s Medium (DMEM, Gibco, Carlsbad, CA, USA) with 10% fetal bovine serum (FBS) (HyClone, Logan, UT, USA), penicillin (10,000 units/ml), and streptomycin (10,000 μg/ml) (Invitrogen). All the cells were maintained in a 5% CO_2_ humidified atmosphere at 37 °C.

### Exosome isolation and incubation with CD8^+^ T cells

To isolate exosomes from serum or cell culture media, the Total Exosome Isolation Kit (from plasma) (Cat#4484450, Invitrogen, USA) and the Total Exosome Isolation Reagent (from cell culture media) (Cat# 4478359, Invitrogen, USA) were used following the manufacturer’s protocol. For incubation of exosomes with CD8^+^ T cells, pre-activated CD8^+^ T cells were seeded on 12-well dishes, and 500 μg exosomes was added into each well. After incubation for 24 h, CD8^+^ T cells were collected for qRT-PCR and the Flow cytometry.

### RNA extraction

Total RNAs were extracted using TRIzol reagent (Takara, Dalian, China) according to the manufacturer’s instruction. The nuclear and cytoplasmic fractions were extracted using PARIS Kit (Ambion, Life Technologies). For the RNAs in exosomes isolation, total RNA of the acquired exosome pellet was extracted using the mirVana PARIS Kit (Ambion, Thermo Scientific, Shanghai, China) according to the manufacturer’s protocol. Synthetic *Caenorhabditis elegans* miRNA cel-miR-39 (5′-UCACCGGGUGUAAAUCAGCUUG-3′), (RiboBio, Guangzhou, China) was spiked into the denatured exosomes as a normalization control. The quality and quantity of isolated RNA was detected by Nanodrop 2000 spectrophotometer (Thermo Fisher Scientific, USA).

### RT-PCR and qRT-PCR

RNA was reverse transcribed using HiScript II Q RT SuperMixfor qPCR ( + gDNA wiper) (Vazyme, Nanjing, China). The AmpliTaq DNA Polymerase (Life Technologies) was used for PCR. The cDNA and gDNA PCR products were observed using 2% agarose gel electrophoresis. For qRT-PCR of circRNAs and genes, the AceQ qPCR SYBR Green Master Mix (Vazyme, Nanjing, China) was used. For miRNAs, the Hydrolysis probe-based qRT-PCR assay was performed according to the manufacturer’s instructions (Applied Biosystems). Primers are listed in Table 2.

**Table 2:** The primers used for qRT-PCR.

Genes	Forward	Reverse
*circRNA-002178*	AGCCCGGGAAGGCGAGACAG	GCTCGGGGGCCCTGTTGG
*RPPH1*	TGGGCAGGAGATGCCGTGGA	CAAAGGAGGCATCCGCCGGG

### RNase R treatment and actinomycin D assay

Two micrograms of total RNA was incubated for 30 min at 37 °C with or without 5 U/μg RNase R (Epicenter Technologies). 95D cells were exposed to 2 μg/ml actinomycin D (Sigma) at indicated time point. Then total RNA of these cells was extracted by TRIzol reagent and analyzed using qRT-PCR.

### Vector construction and cell transfection

For luciferase reporter vector, the sequence of circR-002178, PDL1 3′UTR, PD1 3′UTR, mutated circR-002178, mutated PDL1 3′UTR and PD1 3′UTR was synthesized and then cloned into the downstream of pGL3-promoter (Geneseed, Guangzhou, China). The relative luciferase activity was examined by Dual Luciferase Assay Kit (Promega, Madison, WI, USA). Small interfering RNAs (siRNAs) of circR-002178, miR-34a mimics, miR-28-5p mimics, and corresponding negative control (NC) were synthesized by GenePharma (Shanghai, China). Cells were transfected using Lipofectamine 3000 (Invitrogen) according to the manufacturer’s instruction.

### RNA immunoprecipitation (RIP) and biotin-coupled miRNA capture

The Magna RIP RNA-Binding Protein Immunoprecipitation Kit (Millipore, Billerica, MA) and AGO2 antibody (Argonaute 2 (C34C6) Rabbit mAb #2897, Cell Signaling Technology, Beverly, MA) were used to RIP experiments according to the manufacturer’s instructions. For biotin-coupled miRNA capture, The 3′ end biotinylated miR-34a, miR-28-5p or control RNA were transfected into 95D cells for 48 h before harvest. Then 0.5 ml cell lysis buffer (Invitrogen, USA) wih complete protease inhibitor cocktail (Roche Applied Science, IN) were added into the cell pellets, and incubated on ice for 30 min. The biotin-coupled RNA complex was pulled down by incubating the cell lysates with streptavidin-coated magnetic beads (Life Technologies) by centrifugation at 10,000 × *g* for 20 min.

### Western blot analysis

The total protein of 95D cells were exacted using protein extraction reagent (Thermo Scientific) with a cocktail of proteinase inhibitors (Roche Applied Science, Switzerland). Equal amount of total protein was separated by 10% sodium dodecyl sulfate–polyacrylamide gel electrophoresis and transferred onto a polyvinylidene fluoride membrane. The membranes were blocked with 5% skimmed milk powder and incubated with primary antibodies against PDL1(PD-L1 (E1L3N®) XP® Rabbit mAb #13684, Cell Signaling Technology, Beverly, MA, USA) and GAPDH (GAPDH (D16H11) XP® Rabbit mAb #5174, Cell Signaling Technology, Beverly, MA, USA) at 4 °C overnight and then incubated with secondary antibodies (Anti-rabbit IgG, HRP-linked Antibody #7074, Cell Signaling Technology, Beverly, MA, USA) at room temperature for 2 h. Finally, the bands were examined by Immobilob™ Western Chemiluminescent HRP Substrate (Millipore, Billerica, MA, USA).

### CD8^+^ T isolation

Human CD8^**+**^ T cells purified from healthy donor peripheral blood mononuclearcells (PBMCs) by EasySepTM Direct Human CD8^**+**^ T-Cell Isolation Kit (STEMCELL Technologies). For T-cell activation and proliferation, human CD8^**+**^ T cells were added to anti-CD3/anti-CD28 antibody (BD Biosciences) pre-bound 24-well plates for 48 h. The human T-cell transfection kit (Lonza) was used to transfected the miRNA mimic or scramble RNA into activated CD8^**+**^ T cell.

### Flow cytometry

Flow cytometry was performed using a CytoFLEX S (Beckman Coulter Life Sciences, Mississauga, ON). Human CD8^**+**^ T cell were stained with 0.2 µg of PE anti-PD1 (PE Mouse anti-Human CD279 (PD-1) Clone EH12.1, BD Pharmingen) antibody.

### Expression and statistical analyses

Adequate sample size was determined according to the previous studies that performed analogous experiments. A two-sided test was applied. The raw data applying *t*-test was normally distributed. The variance was similar between the groups that are being statistically compared. Data are expressed throughout the manuscript as mean ± SD. The SPSS 18.0 software was performed to the statistical analyses, and the GraphPad Prism 6.0 (GraphPad Software, San Diego, CA, USA) was used to generate the graphs. The Mann–Whitney *U-*test was used to compare significant differences in exosomal circRNA expression between the LUAD patients and healthy volunteers. Receiver operating characteristic curve (ROC) analysis was utilized to estimate the diagnostic value of exosomal circRNA. A *P*-value < .0.05 was regarded as statistically significant.

## Results

### Characterization of circRNAs in LUAD tissues

**Fig. 1:**
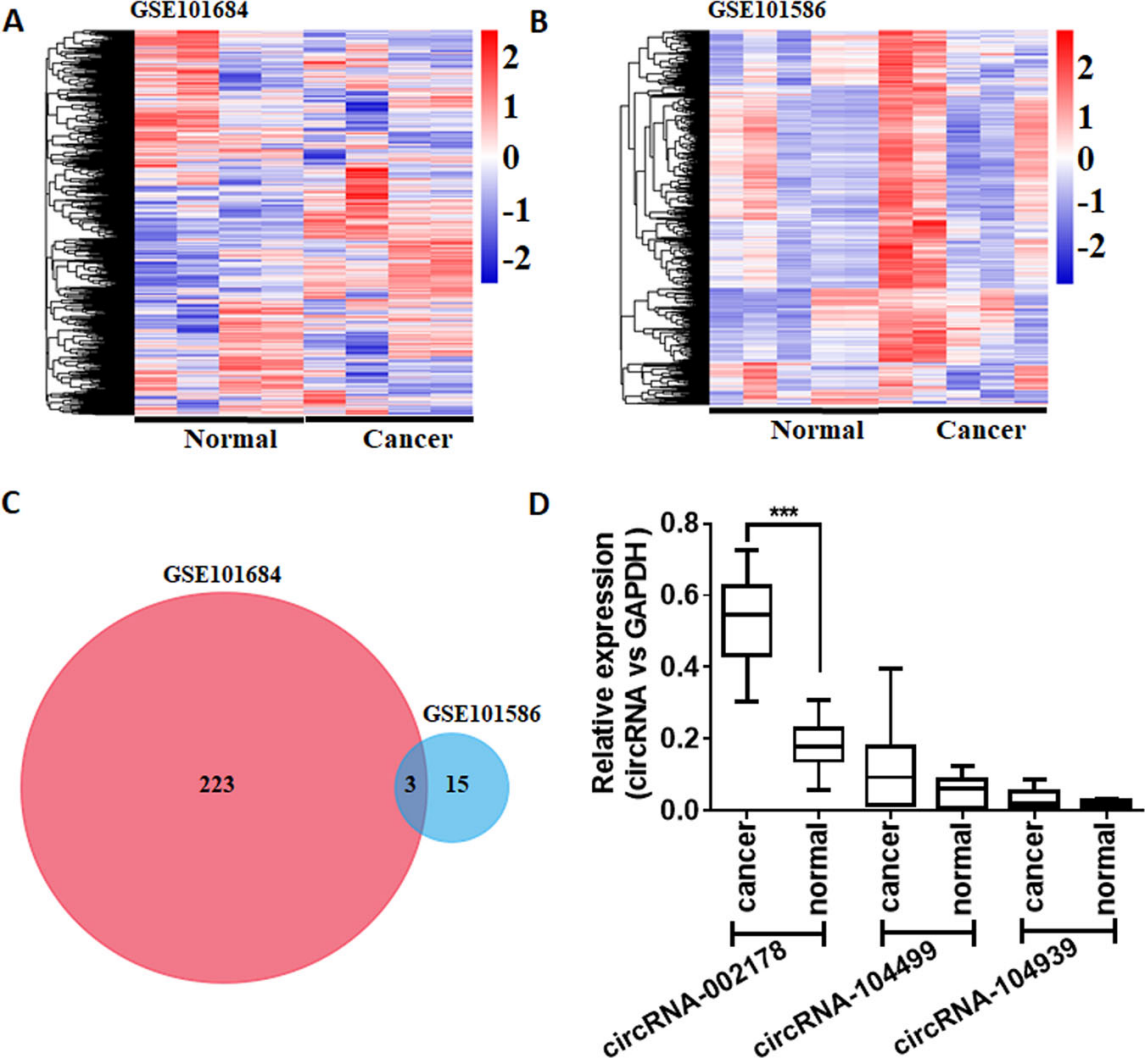
circRNA expression profiles in LUAD. **a**, **b** The heatmap of circRNA profiles in lung adenocarcinoma tissues (Can) and normal lung tissues (Nor). **c** The Venn diagram. **d** The expression level of circRNA-002178, circR-104939, and circRNA-104499 in 20 LUAD cancer tissues and paired adjacent non-cancerous tissues. ***P < 0.001 as determined by two-tailed *t*-test.

Microarray data were collected from two studies on circRNA expression in LUAD vs. paired adjacent non-cancerous tissues. Two-hundred twenty-six circRNAs were identified to be highly expressed in LUAD in the study performed by Zhao et al.^9^ (Fig. 1a), and 18 circRNAs were identified to be highly expressed in LUAD in the study performed by Chen et al.^10^ (Fig. 1b). Three circRNAs, including circRNA-002178, circRNA-104939, and circRNA-104499 were found to be highly expressed in both studies (Fig. 1c). By qRT-PCR assay, only circRNA-002178 was unregulated in LUAD tissues compared to adjacent normal tissues (Fig. 1d).

### CircRNA-002178 was highly expressed in LUAD

**Fig. 2:**
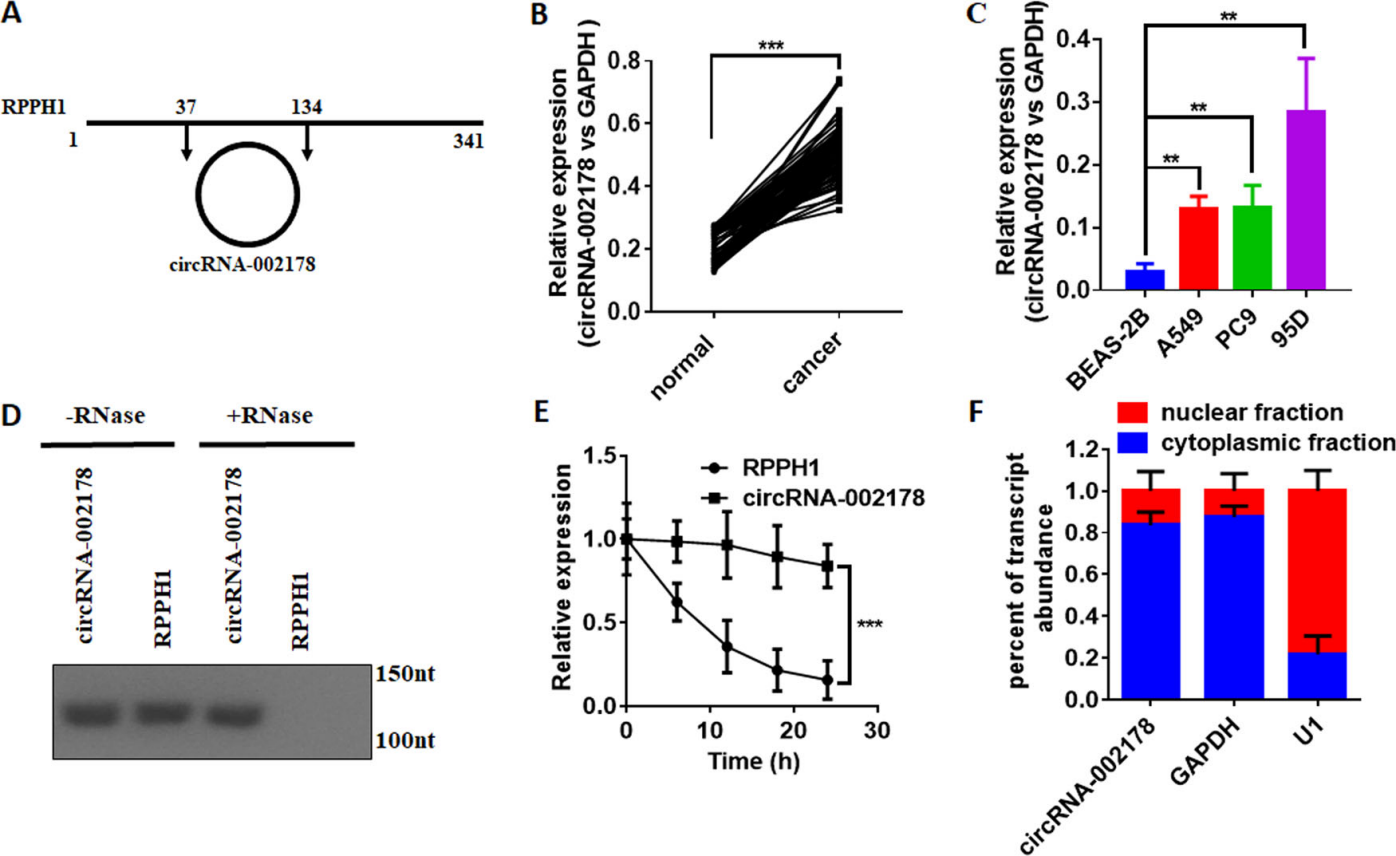
circRNA-002178 was significantly unregulated in LUAD. **a** Genomic loci of circRNA-002178 gene. circRNA-002178 is produced at the RPPH1 gene locus. **b** qRT-PCR for the abundance of circRNA-002178 in 85 LUAD cancer tissues (Cancer) and paired adjacent non-cancerous tissues (Normal). **c** qRT-PCR for the abundance of circRNA-002178 in normal lung cell line BESA-2B and LUAD cancer cell line A549, PC9 and 95D. **d** PCR analysis for circRNA-002178 in cDNA in 95D cells treated with RNase or without. **e** qRT-PCR for the abundance of circRNA-002178 and RPPH1 in 95D cells treated with Actinomycin D at the indicated time point. **f** Levels of circRNA-002178 in the nuclear and cytoplasmic fractions of 95D cells. **P < 0.01 and ***P < 0.001 as determined by two-tailed *t*-test.

CircRNA-002178 derived from a long non-coding RNA, named RPPH1 (Ribonuclease P RNA Component H1) located at chr14:20811436-20811534 with a length of 98 nt (Fig. 2a). We further investigated the expression level of circRNA-002178 in 20 pairs of LUAD tissues by qRT-PCR. In accordance with the data before, the results showed circRNA-002178 were markedly upregulated in LUAD tissues compared with adjacent non-tumor tissues (Fig. 2b). Then, we also observed the expression of circRNA-002178 in three kinds of LUAD cancer cell line (A549, PC9, and 95D) and normal human bronchial epithelial cell line (BEAS-2B). As expected, circRNA-002178 was much higher in cancer cell lines, compared to the normal cell line (Fig. 2c). Subsequently, we performed RT-PCR after RNase R treatment to check the resistance of the circRNA-002178 to RNase R digestion. The results showed only circRNA-002178 was resistant to RNase R treatment compared to RPPH1 linear isoform (Fig. 2d). The stability of circRNA-002178 and RPPH1 were further analysed in 95D cells treated with Actinomycin D, an inhibitor of transcription. As previously reported^3^, We found the circRNA-002178 was extremely more stable than RPPH1 (Fig. 2e). To observe the cellular localization of circRNA-002178, the nuclear and cytoplasmic fraction were isolated by PARIS Kit (Ambion, Life Technologies). As showed in Fig. 2f, circRNA-002178 transcript was preferentially located in the cytoplasm (Fig. 2f).

### circRNA-002178 enhance PDL1 expression via absorbing miR-34a

**Fig. 3:**
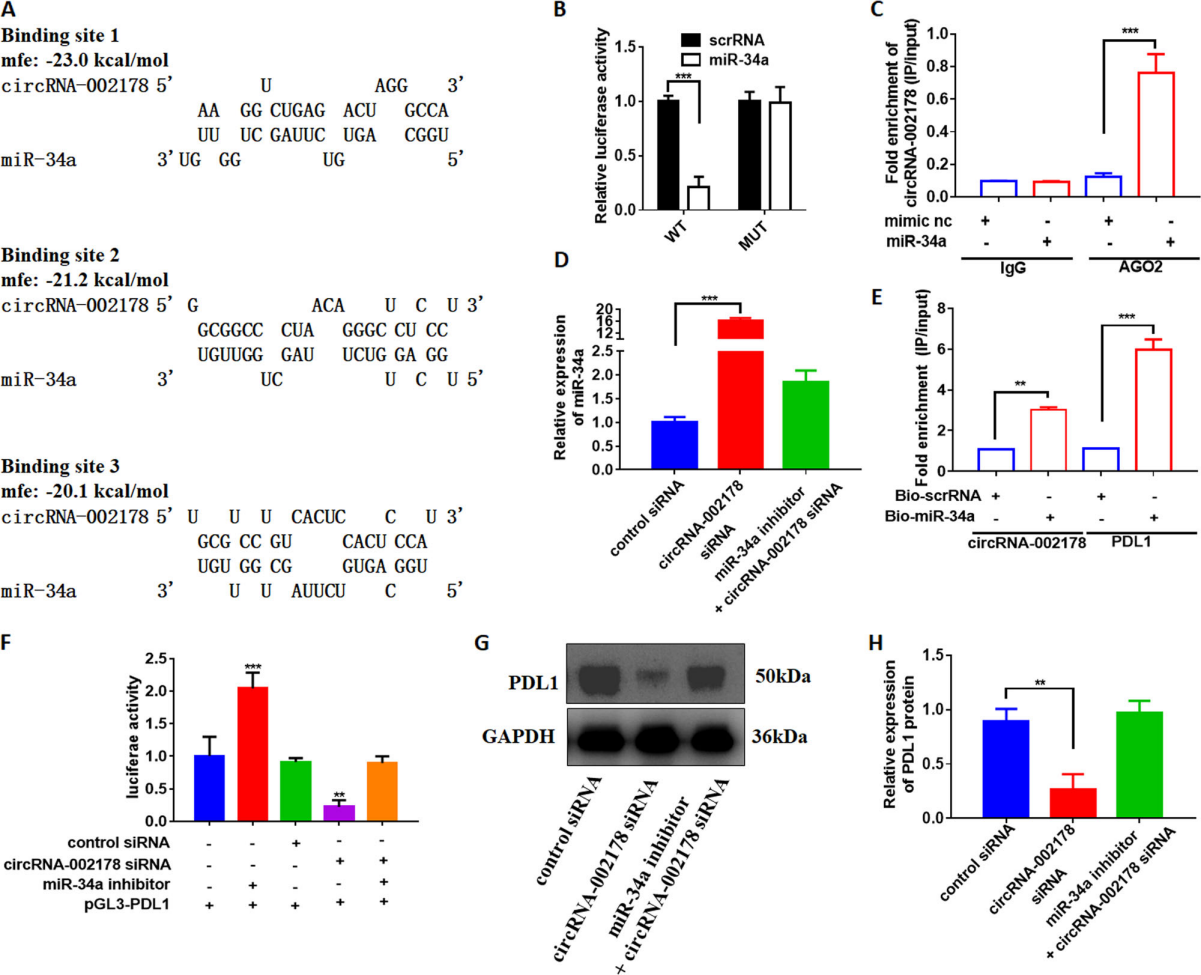
circRNA-002178 promotes PDL1 expression via sponging miR-34a. **a** Schematic descriptions of the hypothetical duplexes formed by miR-34a with circRNA-002178. **b** Luciferase activity of circRNA-002178 in 95D cells transfected with miRNA mimics, which are putative binding to the circRNA-002178 sequence. Luciferase activity was normalized by Renila luciferase activity. **c** RIP was performed using AGO2 antibody in 95D cells transfected with miR-34a mimics or mimics NC, then the enrichment of circRNA-002178 was detected. **d** qRT-PCR for the abundance of miR-34a in 95D cells. **e** circRNA-002178 and PDL1 was pulled down and enriched with 3′-end biotinylated miR-34a in 95D cells. **f** Luciferase reporter activity of PDL1 in 95D cells. **g**, **h** Western blot analysis of PDL1 levels in 95D cells. **P < 0.01 and ***P < 0.001 as determined by two-tailed *t*-test.

CircRNA can act as a ceRNA to absorb microRNA and indirectly stimulate the protein expression of the target genes of miRNAs^11^. As the circRNA-002178 has been found to be remarkably upregulated in LUAD tissues, the miRNAs downregulated in the LUAD tissues were choosesed to predict the potential binding sites in the circRNA-002178 by miRanda^12^ and RNAhydrid^13^. We analyzed the miRNA expression profiles of the LUAD tissues and non-tumor tissues in the TCGA database, and found 30 miRNAs showed significantly downregulated in LUAD cancer tissues (Table. S1). Among these 30 miRNAs, miR-30c-3p, miR-133a-3p, and miR-34a showed to have at least one binding site with circRNA-002178. We further detected the expression level of these three miRNAs in 20 pairs of LUAD tissues by qRT-PCR. Only miR-34a showed significantly decreased in LUAD tissues (Fig. S1). As predicted by miRanda and RNAhydrid, miR-34a have three binding sites with circRNA-002178 (Fig. 3a). To validate the binding capability of the miRNAs to circRNA-002178, the circRNA-002178 luciferase reporter system was constructed and transfected with miR-34a mimics or scramble RNA into 95D cells. The results showed miR-34a significantly reduced the luciferase activity, while has no influence on the mutated vector, which has no binding site of miR-34a (Fig. 3b). Argonaute 2 (AGO2), the key component of RNA-induced silencing complex (RISC), is the major protein in the regulation of miRNA for its target gene. We pulled down the RNA transcripts, which bind to AGO2 with anti-AGO2 antibody by the anti-AGO2 RNA immunoprecipitation (RIP) assay in 95D cells. Compared to the IgG, circRNA-002178 and miR-34a were significantly enrich in the AGP2 pull-down complex (Fig. 3c). These results showed circRNA-002178 could bind with miR-34a in tumor cells. As expected, the miR-34a remarkably increased when the circRNA-002178 was knockdowned by the specific circRNA-002178 siRNAs (Fig. 3d and S2A), while this increase could be abolished by co-transfected with miR-34a inhibitor, which could inhibit miR-34a (Fig. 3h and Fig. S2C). Since it is well known that miR-34a could repress PDL1 expression in cancer cells^14^. We found knockdown miR-34a by miR-34a inhibitor could upregulate PDL1 expression (Fig. S2D). The miRNA pull-down assay with specific biotin-labeled miR-34a also showed circRNA-002178 and PDL1 was specific enriched in the biotin-labeled miR-34a group compared with control (Fig. 3e). Additionally, the activity of luciferase reporter vector carrying the *PDL1 1* 3′UTR sequence could be significantly decreased by knockdown circRNA-002178 through siRNAs, while this decrease could be attenuated by miR-34a inhibitor (Fig. 3f). Finally, we evaluated the protein expression of PDL1 in 95D cells transfected with circRNA-002178 siRNA. Result showed that knockdown circRNA-002178 by siRNA significantly decreased PDL1 expression (Fig. 3g, h). As miR-34a inhibitor could suppress the increase of miR-34a caused by the circRNA-002178 siRNA (Fig. 3d), the decrease of PDL1 induced by circRNA-002178 siRNA could be abolished by miR-34a inhibitor (Fig. 3g, h). Taken together, our result suggested circRNA-002178 could enhance PDL1 expression via absorbing miR-34a in cancer cells.

### circRNA-002178 existed in exosomes and serve as a novel diagnosis biomarker for LUAD

**Fig. 4:**
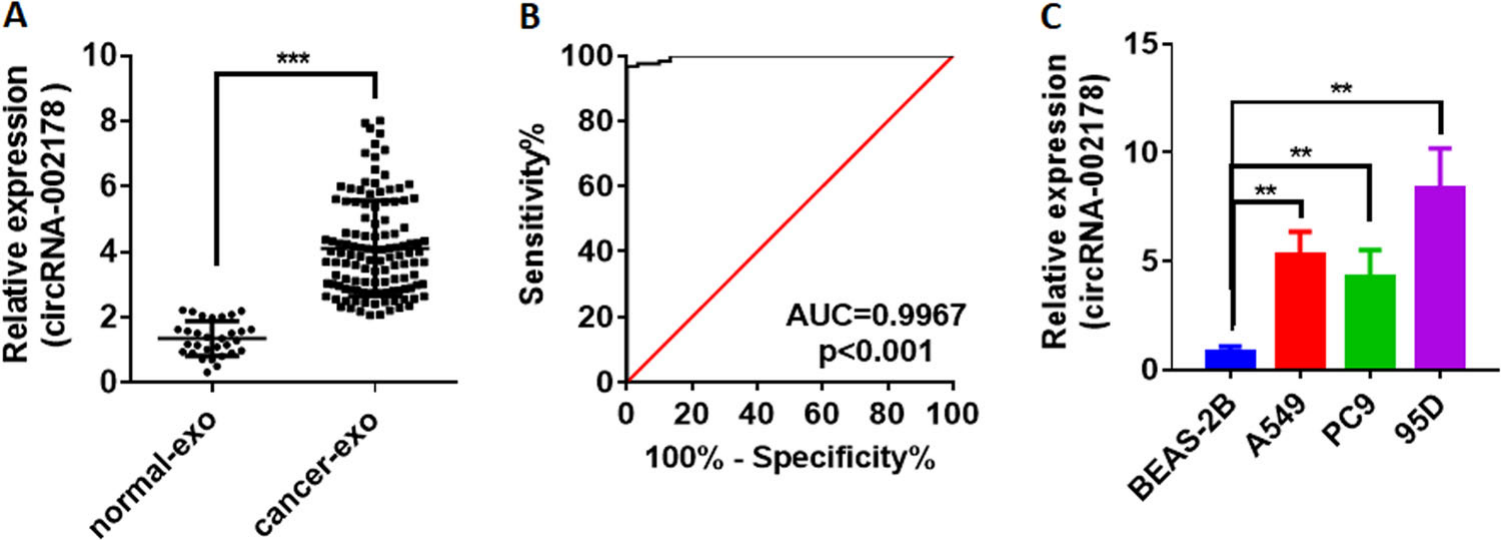
circRNA-002178 existed in exosomes and serve as a novel diagnosis biomarker for LUAD. **a** The expression of circRNA-002178 in the serum exosomes from patients with LUAD (*N* = 120) and normal samples from healthy volunteers (*N* = 30). **b** ROC analysis for the circRNA-002178. **c** the expression of circRNA-002178 in the exosomes derived from normal lung cell line BESA-2B and LUAD cancer cell line A549, PC9, and 95D. **P < 0.01 and ***P < 0.001 as determined by two-tailed *t*-test.

Exosomes, secreted by cancer cells, have been found to contain lots of RNAs, including miRNA, lncRNA, and circRNA^6^. We isolated exosomes in the serum from healthy volunteers or LUAD patients, and performed qRT-PCR to detect the circRNA-002178 in the exosomes. The results showed circRNA-002178 was significantly increased in exosomes from the LUAD patients compared to the healthy volunteers (*P* < 0.001, Fig. 4a). The areas under the curve (AUCs) of the circRNA-002178 were 0.9956 (*P* < 0.001, Fig. 4b). As the results in the patients, we also found the circRNA-002178 was much higher in the exosomes derived from cancer cells than the exosomes derived from normal human bronchial epithelial cells (Fig. 4c). These results reaffirmed that serum exosomal circRNA-002178 could serve as a novel diagnosis biomarker for LUAD. However, this needs to be further studied in the big cohort.

### circRNA-002178 promoted PD1 expression via absorbing miR-28-5p in CD8^+^ T cells

**Fig. 5:**
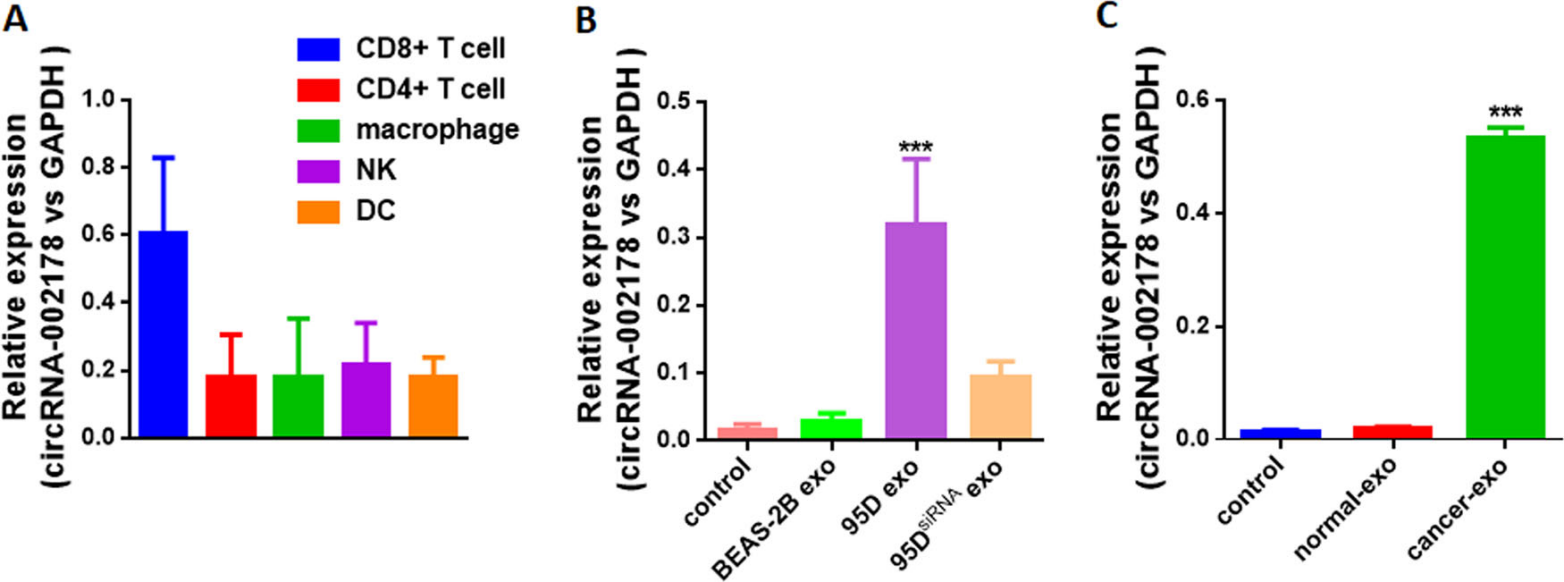
circRNA-002178 could be delivered into CD8^+^ T cells via exosomes secreted by cancer cells. **a** The expression of circRNA-002178 in the immune cells derived from normal tissues or lung cancer tissues. **b** The expression of circRNA-002178 in the CD8^+^ T cells incubated with exosomes derived from BEAS-2B, 95D, or 95D transfected with circRNA-002178 siRNAs. **c** The expression of circRNA-002178 in the CD8^+^ T cells incubated with exosomes derived from healthy volunteer serum or LUAD patient serum. Each value represents the mean ± SD; ***P < 0.001 as determined by two-tailed *t*-test.

In the tumor microenvironment, crosstalk of cancer cells with immune cells is essential for tumor immune invasion^15^. Exosomes derived from cancer cells are involved in tumor immune escape by delivering RNAs (such as miRNAs, circRNAs) into immune cells^6,16^. In order to identify the function of circRNA-002178 in exosomes during the tumor immune escape, we firstly detected the circRNA-002178 expression level in the immune cells isolated from cancer tissues, and found circRNA-002178 significantly enriched in the CD8^+^ T cells (Fig. 5a). Thus, these results suggested circRNA-002178 could be transferred from cancer cells into D8^+^ T cells via exosomes. Then we isolated the exosomes from the culture media of LUAD cancer cell line 95D or normal human bronchial epithelial cell line BEAS-2B, and incubated the exosomes with pre-activated CD8^+^ T cells. As showed in Fig. 5b, the circRNA-002178 markedly increased in the CD8^+^ T cells incubated with the exosomes from 95D cells, compared to the CD8^+^ T cells incubated with the exosomes from BEAS-2B cells (Fig. 5b). Additionally, when the circRNA-002178 in the exosomes derived from 95D cells was knockdowned by the siRNA (Fig. S2B), the circRNA-002178 was found not to be upregulated in the CD8^+^ T cells (Fig. 5b). More interestingly, the exosomes derived from cancer patient serum could also significantly increase the expression level of circRNA-002178 in CD8^+^ T cells, compared to the exosomes from serum of healthy volunteers (Fig. 5c). In summary, these result showed circRNA-002178 in the exosomes secreted by cancer cells could be transferred into CD8^+^ T cells.

**Fig. 6:**
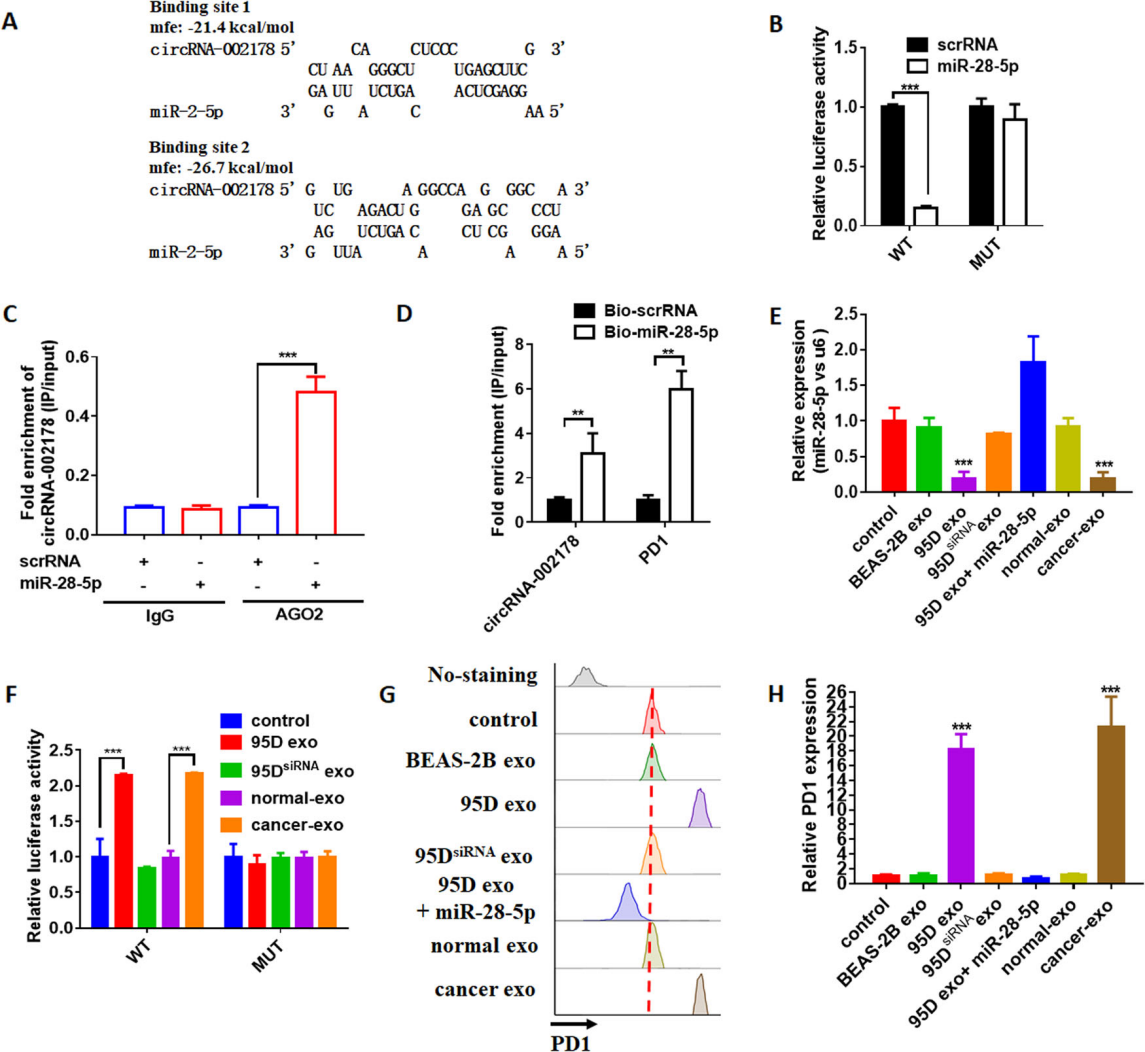
circRNA-002178 relieving the suppression of miR-28-5p for PD1 expression in CD8^+^ T cells. **a** Schematic descriptions of the hypothetical duplexes formed by miR-28-5p with circRNA-002178. **b** Luciferase activity of circRNA-002178 in T cells transfected with miRNA mimics, which are putative binding to the circRNA-002178 sequence. Luciferase activity was normalized by Renila luciferase activity. **c** RIP was performed using AGO2 antibody in T cells transfected with miR-28-5p mimics or mimics NC, then the enrichment of circRNA-002178 was detected. **d** circRNA-002178 and PD1 was pulled down and enriched with 3′-end biotinylated miR-28-5p in T cells. **e** qRT-PCR for the abundance of miR-28-5p in T cells. **f** **l**uciferase reporter activity of PD1 in T cells incubated with exosomes derived from 95D, 95D transfected with circRNA-002178 siRNAs, healthy volunteer serum or LUAD patient serum. **g**, **h** Flow cytometry analysis of PD1 levels in T cells. **P < 0.01 and ***P < 0.001 as determined by two-tailed *t*-test.

As previously mentioned, circRNAs usually regulate the gene expression by acting as miRNA sponges. We use the miRanda and RNAhydrid to predicted the binding site of circRNA-002178 and the dysregulated miRNAs in the activated CD8^+^ T cells^17,18^. The result showed miR-28-5p have two binding sites in the circRNA-002178 (Fig. 6a). In order to confirm the bioinformatics prediction, we transfected the full-length of circRNA-002178-wt and mutant (without miR-28-5p binding sites) luciferase reporter vector pGL3 into CD8^+^ T cells with miR-28-5p mimic or scrRNA. The result showed miR-28-5p mimics could significantly downregulated the luciferase activity of WT group but not mutant one (Fig. 5b). Then, we performed the anti-AGO2 RNA immunoprecipitation (RIP) assay to pull down the RNA transcripts, which bind to AGO2 with anti-AGO2 antibody in CD8^+^ T cells. As showed in Fig. 6c, circRNA-002178 and miR-28-5p were all efficiently pulled down by anti-AGO2 antibodies compared with IgG. These results suggested there might be a direct interaction between circRNA-002178 and miR-28-5p. It has been reported miR-28-5p could repress PD1 expression by binding the 3′UTR of PD1 in CD8^+^ T cells^19^. As the previous reported^19^, we found miR-28-5p could repress the luciferase activity of luciferase reporter vector pGL3 containing the PD1 3′UTR sequence (Fig S3B), and repress the expression of PD1 in CD8^+^ T cells (Fig. S3C, D). Subsequently, the biotin-labeled miR-28-5p was transfected into CD8^+^ T cells to perform the miRNA pull-down assay. The results showed a specific enrichment of circRNA-002178 and PD1 in the biotin-labeled miR-28-5p group compared with control (Fig. 6d). Then, we incubated the exosomes derived from 95D cells or BEAS-2B cells with CD8^+^ T cells. As the exosomes secreted from 95D cells and serum of LUAD patients could increase the expression level of circRNA-002178 in CD8^+^ T cells, we found miR-28-5p significantly decreased in CD8^+^ T cells incubated with these exosomes, compared to the exosomes from BEAS-2B cells and serum of healthy volunteers (Fig. 6e), and the decrease could be attenuated by miR-28-5p mimic (Fig. 6e). Immediately, the luciferase reporter vector pGL3 with the PD1 3′UTR sequence or mutated sequence were transfected into CD8^+^ T cells, which later incubated with the exosomes. As showed in Fig. 6f, the exosomes from 95D cells or serum of LUAD patients could significantly downregulated the luciferase activity, while the exsomes form 95D cells transfected with circRNA-002178 siRNAs has no effect. Finally, we evaluated the protein expression of PD1 in CD8^+^ T cells. Result showed that the PD1 level significantly increased in CD8^+^ T cells incubated with exosomes from cell media of 95D cells or serum of LUAD patients, compared to the exosomes from BEAS-2B cells, or 95D cells transfected with circRNA-002178 siRNA, or the exosomes from serum of healthy volunteers (Fig. 6g, h). Taken together, our result suggested circRNA-002178 delivered via exosomes derived from cancer cells could promote PD1 expression via absorbing miR-28-5p in CD8^+^ T cells.

## Discussion

**Fig. 7:**
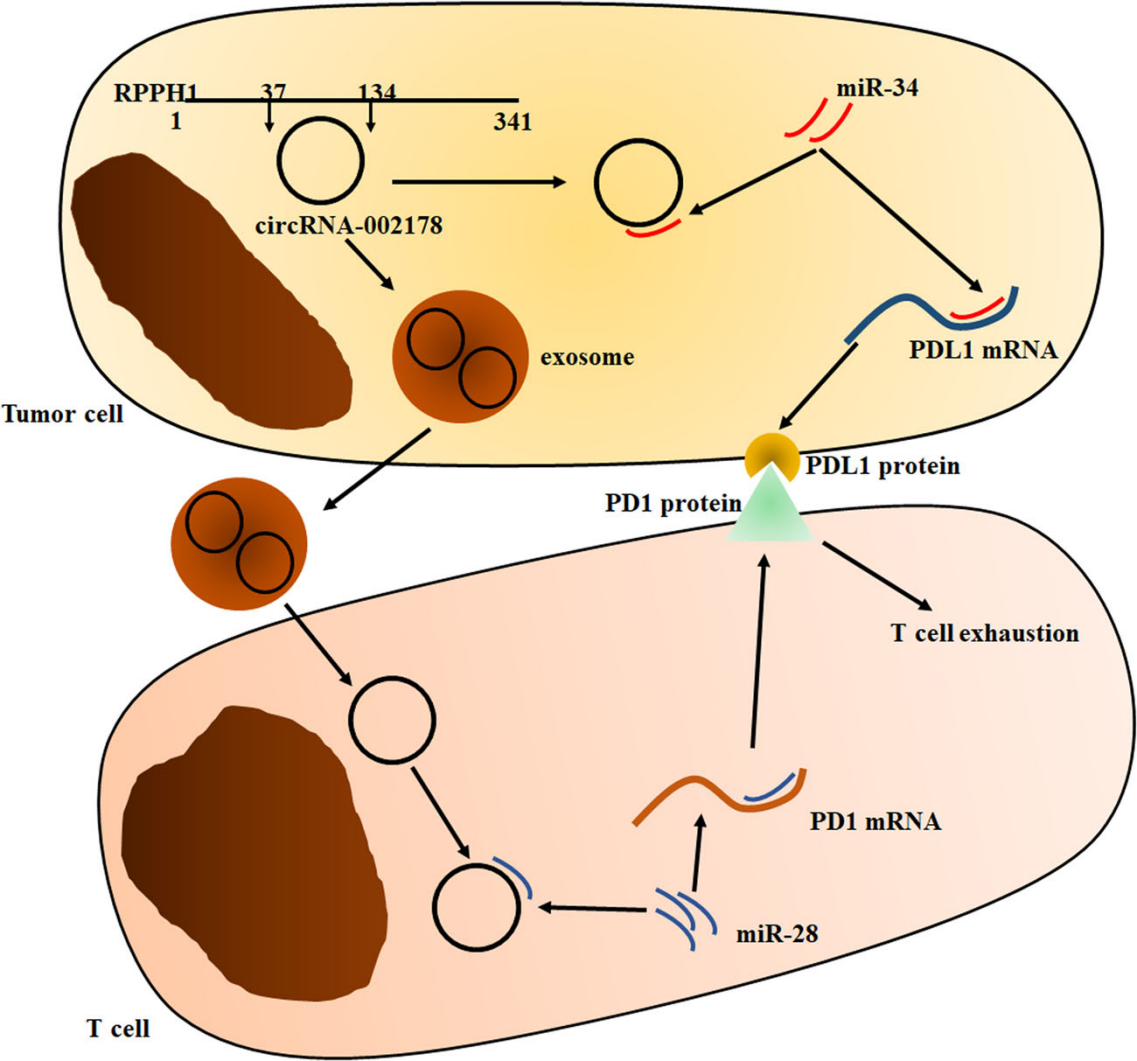
Hypothesis diagram illustrates function and mechanism of circRNA-002178 in LUAD progress.

LUAD is one of the most common malignant tumors worldwide, and its therapeutic effect is still far from satisfactory because of the lack of effective diagnosis and treatment methods^1^. circRNA is novel kind of non-coding RNA by noncanonical splicing without a free 3′ or 5′ end^3^. Numbers of studies have revealed circRNAs play a key role in tumor process^5^. In our study, we found a circRNA named circRNA-002178 significantly increased in the tissues and exosomes, and could have potential to serve as a biomarker for LUAD diagnosis. CircRNA-002178 as one of the 27 circRNAs derived from the exon region of the derived from Ribonuclease P RNA component H1 (RPPH1) has been reported overexpressed in esophageal cancer cells^20^. By function assay, we found circRNA-002178 could act as a sponge for miR-34a and miR-28-5p to relieve the suppression of these two miRNAs for their target genes PDL1 and PD1 in cancer cells and CD8^+^ T cells, respectively (Fig. 7).

The T-cell-based immune system has been confirmed to closely involve in the cancer cell reorganization and clearance^7^. This progress is controlled by a series of co-inhibitory receptors and their ligands (also known as immune checkpoints), which could result to tumor immune escape^7^. Among all the immune checkpoints, the PDL1/PD1 has been proven as a therapeutic target in a large number of malignancies^7^. Recently, antibodies targeting the PDL1/PD-1 have been successfully approved for cancer treatment, including melanoma, lung cancer, renal cell carcinoma, bladder cancer, head and neck squamous cell carcinoma^8^. However, only a minority of patients was found to benefit from the PDL1/PD1 blockade^8^. Several studies have confirmed the expression of PDL1 on tumor cells has been related to the clinical response. Thus, a better understanding of the processes that regulate PDL1/PD1 expression is urgently needed for PDL1/PD-1 therapy. During the past years, the expression of PDL1 in tumor cells is found to be affected by genomic aberrations, inflammatory signaling, oncogenic signaling, posttranslational modulation and microRNA-based control^20^. MiR-34a is a member of miR-34 family and found to be frequently downregulated and could function as tumor suppressive miRNA in solid malignancies^21^. It could directly bind with the 3′UTR of PDL1 and inhibit the expression of this immune checkpoint molecule in many tumors, including LUAD^14^. In our study, we firstly identified a LUAD-specific circRNA, circRNA-002178 could act as sponge for miR-34a to relive the repression of miR-34a for PDL1 and promote PDL1 expression in tumor cells. Activated T cells were found to be high expressed several miRNAs^19^. Among them, miR-28-5p was revealed to bind with the 3′UTR of PD1 to control the expression level of PD1 on T cells^19^. It’s well known that tumor cells and tumor microenvironment could induce PD1 expression on activated T cells to induce T-cell exhaustion. However, the mechanism remains unknown. In our study, we found cancer cells could secrete exosomes, which contains circRNA-002178 and these exosomes could deliver circRNA-002178 into T cells to enhance PD1 expression by sponging the miR-28-5p. As we know, this is the first study to reveal the circRNA derived from tumor cells, which could simultaneously regulate PDL1 and PD1 in tumor cells and T cells via acting as a sponge for miRNAs, respectively (Fig. 7).

To conclude, we found circRNA-002178 was significantly upregulated in the LUAD tissues, cells and exosomes of cancer cells and serum from patients. circRNA-002178 could enhance PDL1 expression via sponging miR-34 in cancer cells. Moreover, circRNA-002178 could also be delivered into T cells to promote PD1 expression via sequestering miR-28-5p through exosomes secreted by cancer cells. Additionally, the exosomal circRNA-002178 significantly upregulated in the serum from LUAD patients. Our results suggested that circRNA-002178 could be used as a potential non-invasive biomarker for the LUAD detection and could act as a target of immune therapy since it could promote the expression of PDL1 and PD1 expression.

## Supplementary information

Supplementary Figure and table Legends

figure S1

figure S2

figure S3

figure S4

table s1

table s2

## Data Availability

All data generated or analyzed during this study are included in this published article. Raw and processed data are stored in the laboratory of global data bank (GB) and are available upon request.
