# Supplementary material for: circRNA-002178 act as a ceRNA to promote PDL1/PD1 expression in lung adenocarcinoma
Source: Cell Death Dis. 2020 Jan 16;11(1):32. doi: 10.1038/s41419-020-2230-9 (PMC6965119; doi:10.1038/s41419-020-2230-9)
Supplement: Supplementary file 1 — Supplementary Figure and table Legends [file 41419_2020_2230_MOESM1_ESM.docx]

**Figure S1. the expres**sion **of miR-30c-3p, miR-133a-3p and miR-34a in the 20 pairs of LUAD tissues by qRT-PCR. ***P<0.001 as determined by two-tailed *t*-test.**

**Figure S2. MiR-34a repress PDL1 expression. (A)** the expression of circRNA-002178 in the 95D cells transfected with circRNA-002178 siRNA. **(B)** the expression of circRNA-002178 in exosomes derived from 95D cells transfected with circRNA-002178 siRNA. **(C)** the expression of miR-34a in the 95D cells transfected with miR-34a inhibitor. **(D)** the expression of PDL1 in the 95D cells transfected with miR-34a inhibitor. Each value represents the mean±SD; *P <0.05 and ***P <0.001 as determined by two-tailed *t-test*.

**Figure S3. miR-28-5p repress PD1 expression. (A)** the expression of miR-28-5p in T cells transfected with miRNA mimics. **(B)** Luciferase activity of PD1 in T cells transfected with miRNA mimics which are putative binding to the PD1 sequence. Luciferase activity was normalized by Renila luciferase activity. **(C-D)** Flow cytometry analysis of PD1 levels in T cells. Each value represents the mean±SD; ***P <0.001 as determined by two-tailed *t-test*.

**Figure S4. Raw gel for figure 2, 3 and S2.**

**Table S1. The downregulated miRNA in LUAD tissues, compared to non-cancerous tissues in TCGA database (fold change <0.05, *p*<0.001).**

**Table S2. Raw data.**
