# Supplementary figures and images for: circRNA-002178 act as a ceRNA to promote PDL1/PD1 expression in lung adenocarcinoma
Source: Cell Death Dis. 2020 Jan 16;11(1):32. doi: 10.1038/s41419-020-2230-9 (PMC6965119; doi:10.1038/s41419-020-2230-9)

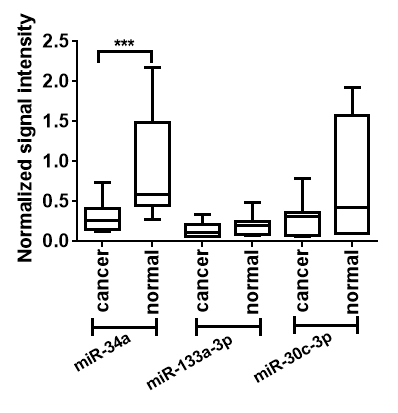

Supplement: Supplementary file 2 — figure S1 [file 41419_2020_2230_MOESM2_ESM.tif]

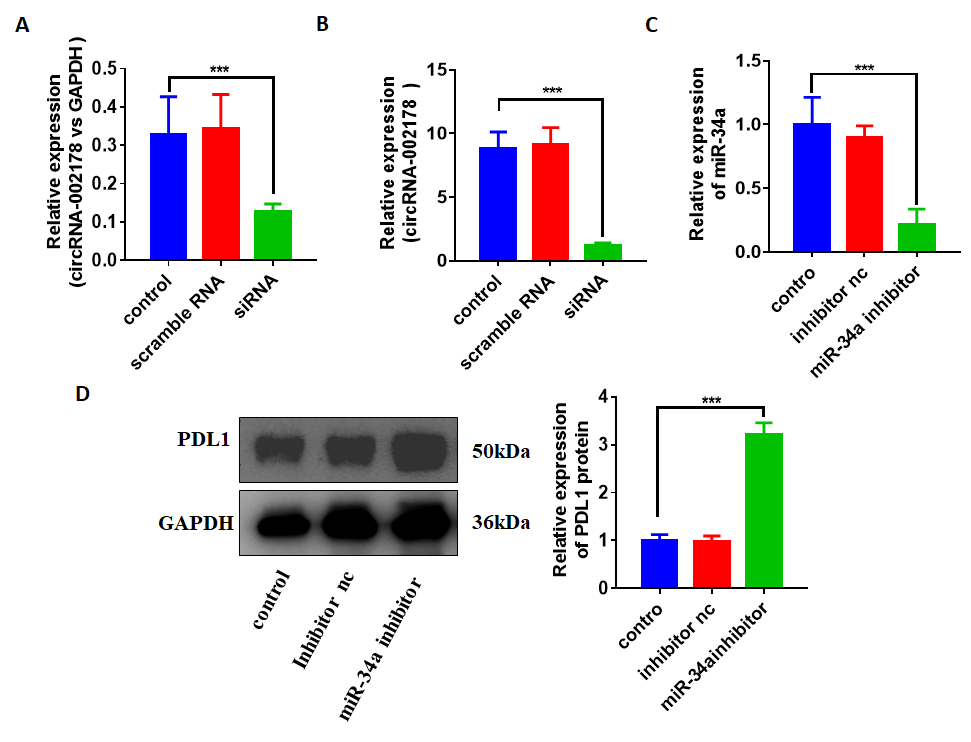

Supplement: Supplementary file 3 — figure S2 [file 41419_2020_2230_MOESM3_ESM.tif]

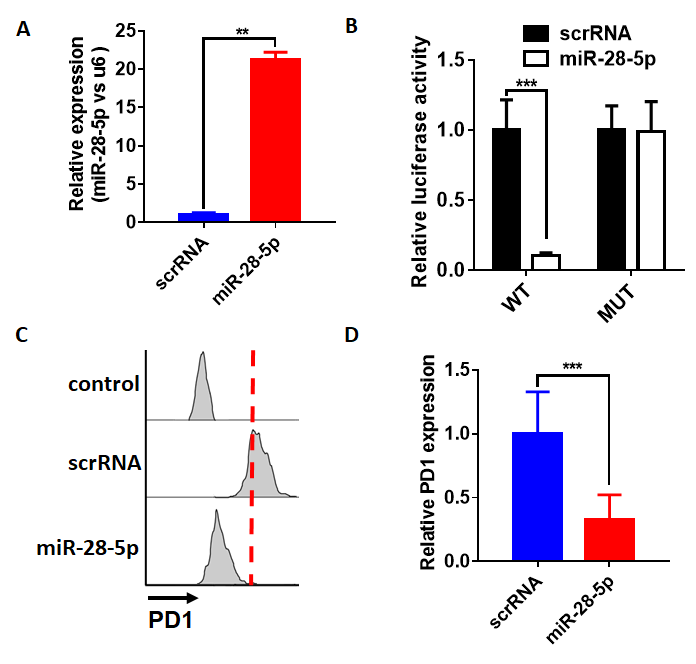

Supplement: Supplementary file 4 — figure S3 [file 41419_2020_2230_MOESM4_ESM.tif]

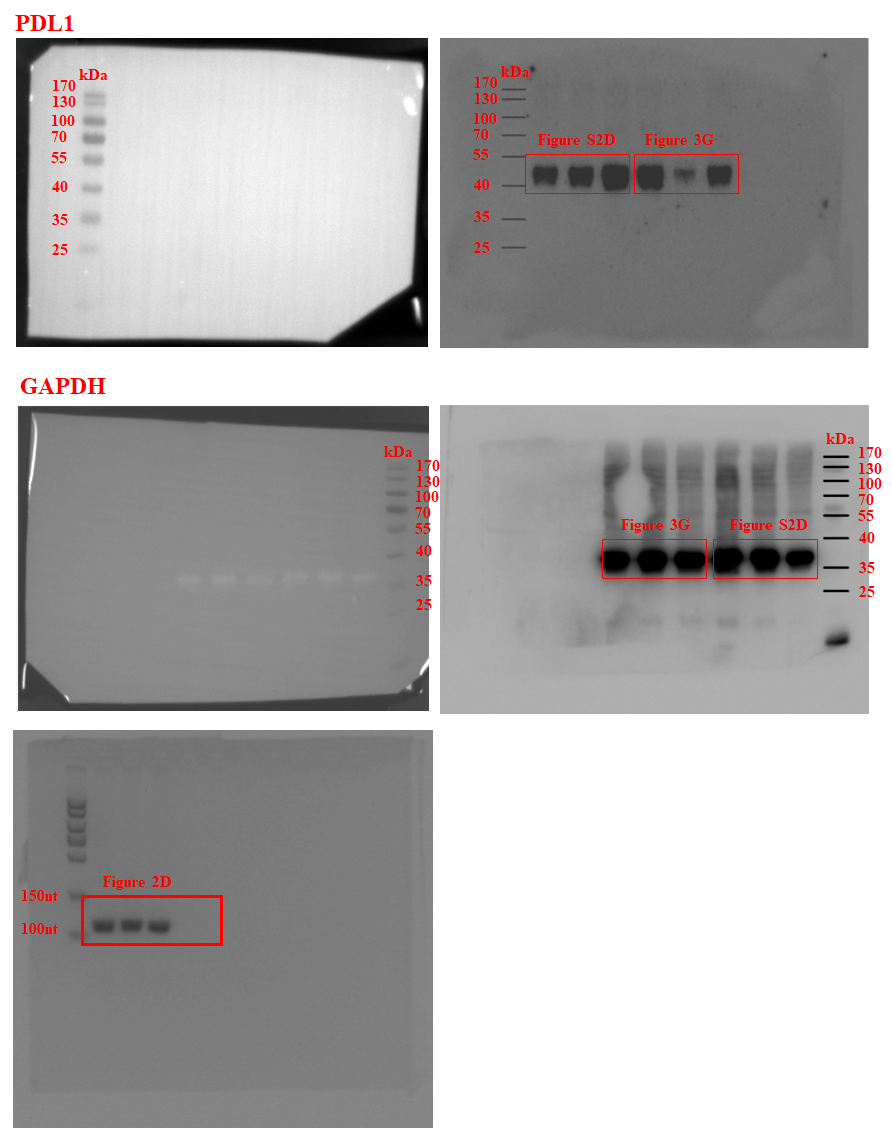

Supplement: Supplementary file 5 — figure S4 [file 41419_2020_2230_MOESM5_ESM.tif]
